# Directly monitor protein rearrangement on a nanosecond-to-millisecond time-scale

**DOI:** 10.1038/s41598-017-08385-0

**Published:** 2017-08-18

**Authors:** Eric H.-L. Chen, Tony T.-Y. Lu, Jack C.-C. Hsu, Yufeng Jane Tseng, T.-S. Lim, Rita P.-Y. Chen

**Affiliations:** 1 Institute of Biological Chemistry, Academia Sinica, Taipei, 115 Taiwan; 2 Institute of Biochemical Sciences, National Taiwan University, Taipei, 106 Taiwan; 3 Department of Computer Science and Information Engineering, National Taiwan University, Taipei, 106 Taiwan; 4 Graduate Institute of Biomedical Electronics and Bioinformatics, National Taiwan University, Taipei, 106 Taiwan; 5 Department of Physics, Tunghai University, Taichung, 407 Taiwan

## Abstract

In order to directly observe the refolding kinetics from a partially misfolded state to a native state in the bottom of the protein-folding funnel, we used a “caging” strategy to trap the β-sheet structure of ubiquitin in a misfolded conformation. We used molecular dynamics simulation to generate the cage-induced, misfolded structure and compared the structure of the misfolded ubiquitin with native ubiquitin. Using laser flash irradiation, the cage can be cleaved from the misfolded structure within one nanosecond, and we monitored the refolding kinetics of ubiquitin from this misfolded state to the native state by photoacoustic calorimetry and photothermal beam deflection techniques on nanosecond to millisecond timescales. Our results showed two refolding events in this refolding process. The fast event is shorter than 20 ns and corresponds to the instant collapse of ubiquitin upon cage release initiated by laser irradiation. The slow event is ~60 μs, derived from a structural rearrangement in β-sheet refolding. The event lasts 10 times longer than the timescale of β-hairpin formation for short peptides as monitored by temperature jump, suggesting that rearrangement of a β-sheet structure from a misfolded state to its native state requires more time than *ab initio* folding of a β-sheet.

## Introduction

**Figure 1:**
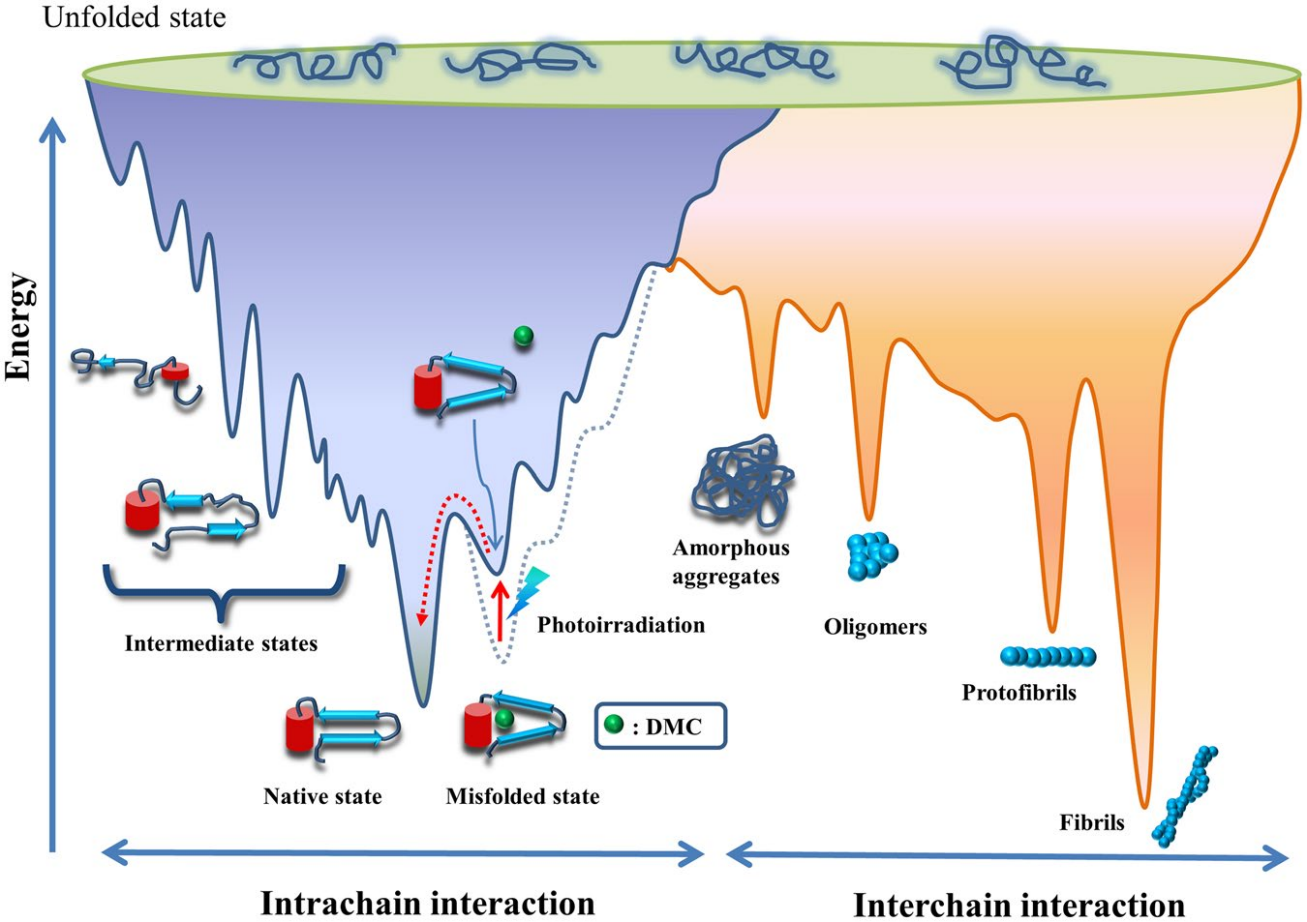
The energy landscape of protein folding and misfolding diagram. In this study, a photolabile cage, which is shown as a green ball, is used to trap a protein in a locked misfolded state and change energy landscape of folding (dashed line). After laser irradiation, the cage is photolyzed and released. The misfolded state is free to refold to the native state.

Protein folding is an important issue in protein science. Figure 1 depicts a protein folding/misfolding funnel model^1, 2^. An unfolded protein folds into its native structure via pathway(s) that are determined by its primary structure and solvent conditions. The on-pathway folding intermediates occur transiently and may not be detectable using current techniques. Typically, the unfolding free energy of a protein is not high (5 to 10 kcal/mol), so a small portion of protein molecules might be partially unfolded (also considered misfolded), with the proportion depending on the solvent conditions. In conditions favoring the population of this partially unfolded state, such as acidic pH, moderate GdnHCl concentrations, or elevated temperatures, these partially unfolded molecules tend to self-associate (probably via the exposed hydrophobic patches on their surface) and assemble into amorphous aggregates, oligomers, or amyloid fibrils^3^. The misfolding process occurs easily in proteins with lower structural stability. For example, a disulfide bond stabilizes prion protein. Without the disulfide bond, the second α-helix of prion protein is unfolded at room temperature, neutral pH, and in the absence of denaturant, and this partially unfolded prion protein gradually assembles into β-oligomers or fibrils^4, 5^. The misfolding kinetics is driven by hydrophobic interaction and can be tuned by salt concentration in the protein solution.

To understand how proteins fold, one common approach is using site-directed mutagenesis to explore the role of individual residues in protein stability and folding kinetics^6–8^. Another common approach is using short synthetic peptides corresponding to the target structural segment to study how local secondary structure forms in the early stage of folding, which is situated at the top of the funnel model of protein folding (Fig. 1). Folding kinetics can be monitored by various spectroscopies combining different unfolding/refolding methods with different observation time windows, for example, stopped flow (>10^−3^ s)^9^, continuous flow mixing (10^−5^~10^−3^ s)^10^, flash photolysis (>10^−9^ s)^11^, pressure jump (>10^−6^ s)^12^, and temperature jump (>10^−9^ s)^13, 14^. It is known that the folding timescale of a tertiary structure generally ranges from a few microseconds to a few seconds^14, 15^. The α-helix is often considered the nucleation site or first step of protein folding, and the folding timescale for the α-helix ranges from a few nanoseconds to hundreds of nanoseconds^16–18^. β-Sheet folding is a popular topic of investigation for its strong stability arising from extensive, long-ranged inter-chain interactions and its implication in misfolding-associated diseases. The residues that preferentially form β-sheets are often aromatic and hydrophobic, and thus prone to aggregation^19^. β-Sheets fold from hundreds of nanoseconds to a few microseconds, more slowly than α-helices^14, 16, 17, 20^. Two mechanisms, the zipper model and the hydrophobic collapse model, have been proposed for β-sheet formation^21^. The zipper model suggests that turn formation is the first step in β-sheet folding, as it directs β-strand orientation^16, 20, 22–26^, whereas the hydrophobic collapse model emphasizes the importance of hydrophobic interactions in proteins and the entropy increase of water molecules in driving protein folding^27^, While most researches focus on the refolding/unfolding process of β-sheet peptides, the results from the β-sheet peptides are not necessarily the same as the folding behavior of the β-sheet within a protein, in which the interactions between the β-sheet and the neighboring residues can affect the process.

For multiphase kinetics of protein folding, the fast event is usually assigned as the folding nucleus formation, and the slow event is structural assembly and rearrangement. When proteins mistakenly fold as off-pathway intermediates, some can refold back to their native state on their own, while the others require assistance from chaperones. It is difficult to directly detect and measure how fast the misfolded intermediates refold because the initial misfolded state is difficult to create. Here, we employed the“caging” technique to trap proteins in a misfolded molten globule state (with native-like secondary structure content but without the tightly packed protein interior) and monitor the structural rearrangement process of ubiquitin from the artificially created misfolded state to the native state (Fig. 1). Our experimental design allows direct observation of the structural rearrangement event into the tertiary structure.

In this study, we used ubiquitin as our study system and 4-(bromomethyl)*-*6,7*-*dimethoxycoumarin (BrDMC) as a structural cage to disturb the ubiquitin structure. We selected Val5 as the caging site because it is situated on the first β-strand and its side-chain faces inward. Because BrDMC reacts with the sulfhydryl group of cysteine, we mutated Val5 → Cys in ubiquitin. BrDMC was labeled to the mutated protein V5C in a partially denaturing condition to produce the caged protein denoted as V5C-DMC. Due to steric hindrance of the DMC moiety, V5C-DMC is misfolded. The misfolded (or partially unfolded) structure was generated by molecular dynamics (MD) simulation. Photocleavage of the cage was initiated by pulse laser, and then the resulting volume change from the heat release/absorption in the cage release event and the consequent ubiquitin refolding events could be recorded by time-resolved photoacoustic calorimetry (PAC) and photothermal beam deflection (PBD)^16, 28–31^. PAC can detect fast events in the time range of 20 ns to 2 μs, and PBD can detect slower events in the range of 2 μs to 1 ms. We were able to solve the refolding process of ubiquitin from the misfolded state to the native state by combining the caging strategy, laser flash photolysis, PAC, and PBD.

## Materials and Methods

### Protein expression and purification

The human ubiquitin gene was cloned into the pET11b vector with a six His-tag at the C-terminus, and the F45 → W mutation was included by site-directed mutagenesis, facilitating protein purification and detection (increasing extinction coefficient of the protein). Val-5 was mutated to Cys for BrDMC coupling, and the mutated protein was denoted as V5C. V5C was over-expressed in *E. coli* BL21 Star (DE3) cells (Invitrogen) in tryptone-phosphate medium (17.6 mM Na_2_HPO_4_, 7.4 mM KH_2_PO_4_, 137 mM NaCl, 2% tryptone, 1.5% yeast extract, pH 7.4) containing filter-sterilized 0.2% glucose and 50 μg/mL of ampicillin. The cells were grown at 37 °C with vigorous shaking (250 rpm) for 3 h (O.D. @600 nm = 0.6), and then V5C expression was induced by adding IPTG to a final concentration of 1 mM with continued incubation for 5 h before the cells were harvested by centrifugation at 3000 g at 4 °C for 30 min. The cell pellet (about 24 g from 2.4 L of culture) was resuspended in 120 mL of cell lysis buffer (40 mM Tris, 150 mM NaCl, pH 8.0), and the cells were lysed by the addition of 0.5 × CellLytic B (Sigma), 0.15 mg/mL of lysozyme, 25 μg/mL of DNase I, 7 mM MgCl_2_, and 1 mM PMSF. The lysate was centrifuged at 15,000 rpm at 4 °C for 60 min. The supernatant was applied to a column pre-packed with Ni^2+^-charged Chelating Sepharose Fast Flow gel (GE healthcare, USA), which was then washed with 5 column volumes of washing buffer (40 mM Tris, 500 mM NaCl, 40 mM imidazole, pH 8.2), and V5C was eluted with 4 column volumes of elution buffer (40 mM Tris, 200 mM NaCl, 500 mM imidazole, 20 mM EDTA, pH 7.2). V5C was concentrated by ultrafiltration (Amicon YM-3 Centriplus filter, GE Healthcare) at 3000 g at 4 °C to a final volume of 5 mL, which was then purified by the size exclusion Superdex-75 column (1.6/60) equilibrated with the SEC buffer (20 mM Tris, 50 mM NaCl, 1 mM KCl, 0.2 mM EDTA, pH 8.2) at a flow rate of 1 mL/min. The eluted V5C was dialyzed against deionized (DI) water overnight at 4 °C and concentrated by ultrafiltration. The purity of the protein was evaluated by SDS-PAGE and protein identification was done by MALDI (matrix-assisted laser desorption ionization) mass spectroscopy.

### DMC-labeling

For the caging strategy, V5C was labeled with Br-DMC (4-(Bromomethyl)-6,7-dimethoxycoumarin). V5C protein solution was diluted into 500 μL of 100 mM Tris and 2 M GdnHCl (pH 8.9) to a final protein concentration of 0.4 mM, and then reacted with 500 μL of 4 mM BrDMC (Tokyo Chemistry Industry Co.) in dimethylformamide for 30 mins at RT in the dark. Reversed-phase HPLC was used to purify the labeling product V5C-DMC.

### Simulation methods

The V5 → C and F45 → W mutations were done on the crystal structure of ubiquitin (PDB: 1UBQ) by Chimera^32^. Partial unfolding (see Supplementary methods) of V5C was done in GROMACS 5.0.2^33^ using AMBER99SB-ILDN force field^34^ to expose the V5 → C site for DMC conjugation. When V5 → C site was solvent-exposed enough to accommodate a DMC molecule, the structure was snapshotted and conjugated with DMC using Chimera. The force field parameters of DMC were calculated using AmberTool 14^35^ (see Supplementary methods). The DMC-conjugated structure was first partially refolded with MD simulation using a simulated annealing method where the system was heated from 25 °C (298 K) to 227 °C (500 K) within 200 ps and cooled to 25 °C (298 K) within 300 ns. The stable N-terminal core (residues M1-E16) obtained from simulated annealing as well as the original V5C structure served as the templates for homology modeling with Modeller 9.14. The DMC-conjugated structure refolded by homology modelling was further relaxed in MD simulation at 25 °C (298 K) for 20 ns. The secondary structure contents of the last 5 ns trajectory were analyzed using GROMACS built-in DSSP^36^ program and validated against experimental datasets.

### Circular dichroism spectroscopy

The protein samples were dissolved in deionized water, and the far-UV and near-UV CD spectra were recorded on a J-815 spectrometer (Jasco, Japan). The bandwidth was set at 2 nm and the step resolution at 0.05 nm. Spectra were averaged from 2 scans. The thermal denaturation curves of the protein samples were recorded at 208 nm from 30 °C to 95 °C, and the temperature increment step was 0.1 °C. The protein concentrations and pH values of the solutions are indicated in the figure legend.

### Protease-resistance assay

The protein samples (10 μg) were reacted with or without different concentrations of trypsin (final enzyme concentration 10 or 100 μg/mL) in 0.5 mM NaOAc (pH 7) and analyzed by 16% SDS-PAGE.

### Nuclear magnetic resonance (NMR) spectroscopy

Samples were prepared by dissolving the proteins in 300 μL of unbuffered 9:1 H2O:D2O. For two-dimensional NMR experiments, the concentration of V5C and V5C-DMC was 175 μM and 90 μM, respectively. The pH values were adjusted to 5.8 without correction for the D/H isotope effect. All spectra were recorded at 295 K. One-dimensional^1^H NMR spectra were recorded on a Bruker AV 800 spectrometer (Bremen, Germany) and WATERGATE W5 pulse sequence was used to attenuate residual water. Two-dimensional total correlated spectroscopy (2D-TOCSY) and two-dimensional nuclear Overhauser effect spectroscopy (2D-NOESY) were recorded on a Bruker AV III 600 spectrometer (Bremen, Germany) with solvent suppression using 3-9-19 pulse sequence. We used a 60 ms mixing time in TOCSY and a 125 ms mixing time in NOESY. Two-dimensional spectra were acquired with 2048 data points in the direct dimension and 256 and 128 increments in the indirect dimension for V5C and V5C-DMC, respectively. Scan number is 80 and 1024 for V5C and V5C-DMC, respectively. Data were processed using Topspin3.1 (Bruker BioSpin) and analyzed using Sparky^37^.

### Time-resolved photothermal methods

**Figure 2:**
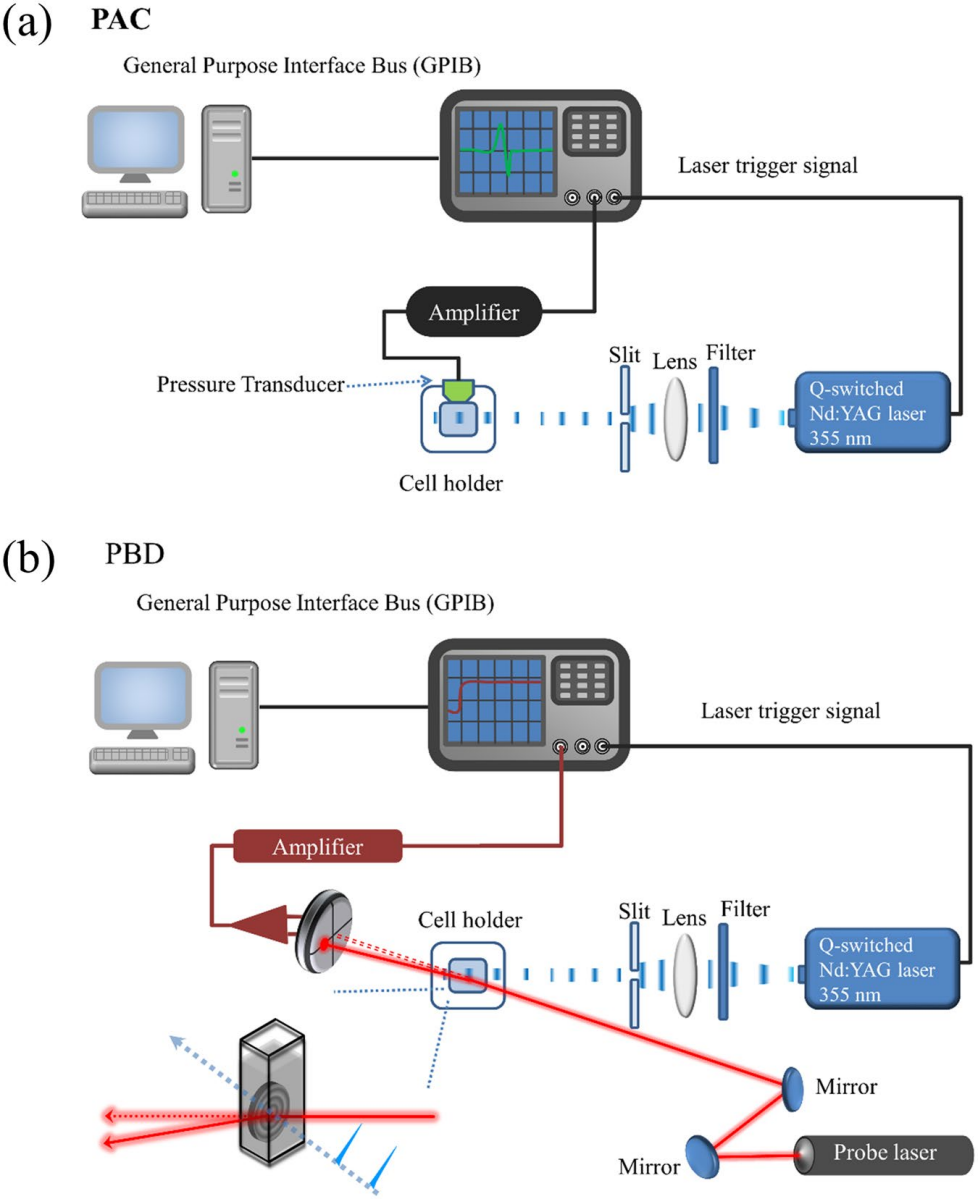
The schematic diagrams of two photothermal methods. (**a**) photoacoustic calorimetry; (**b**) photothermal beam deflection.

V5C-DMC was dissolved in deionized water, and the absorbance of the protein solution at 355 nm was 0.2-0.3 (around 18~27 μM). The time-resolved photothermal method includes the time-resolved photoacoustic calorimetry (PAC) and time-resolved photothermal beam deflection (PBD). The time-resolved PAC system and the time-resolved PBD system are shown in Fig. 2. The light source, operated at 355 nm (the laser pulse width was 5 ns, and the repetition rate was 2 Hz), was the third harmonic of a Q-switch Nd-YAG laser (New Wave Research, Fremont, USA). The time-resolved PAC and PBD signals were measured at 15, 20, 25, 30, and 35 °C, and the temperature variation during an experiment was less than 0.5 °C. The data were analyzed by comparing with those obtained from the calorimetric reference compound, potassium dichromate (K_2_Cr_2_O_7_), which releases all the energy absorbed upon photoexcitation as heat with a quantum efficiency of 1.0^38^. The absorbance at 355 nm of the reference solution was adjusted to be the same as the sample solution. For PAC, the photoacoustic pressure waves were detected using a 1 MHz bandwidth microphone (GE Panametrics V-103, USA). For PBD, the probe laser is He-Ne laser and laser beam deflection is measured by using the quadrant photodiode. The time-resolved PAC and PBD signals from 300 shots were averaged and recorded using a 1 GHz digital oscilloscope. The instrument response time is about 20 ns for PAC and 2 μs for PBD. The time-resolved PAC and PBD signals are a convolution of the instrumental response function (*R*(*t*)) and a function representing the time evolution (*S*(*t*)) of the heat upon laser excitation. The total signal from the sample (*O*(*t*)), which was recorded by oscilloscope, can be written as *O*(*t*) = *R*(*t*)*S(*t*). The signal for the reference compound was used as the instrument response time function *R*(*t*), since the heat release of the reference compound is faster than the instrument response time. *S*(*t*) is often written as the summation of the single exponential terms [see Eq. (1)].

1 $$S(t)=\sum _{i}\frac{{\varphi }_{i}}{{\tau }_{i}}{e}^{\frac{-t}{{\tau }_{i}}}$$

where *ϕ*_*i*_ and *τ*_*i*_ are the respective amplitude and decay time for the *i*th component in the sum of the exponentials. The software program “Sound analysis 32” (Quantum Northwest Inc. USA) was used to fit the data. The program is based on the Least Squares Iterative Reconvolution (LSIR) method.

## Results

### Structural comparison of V5C and V5C-DMC

**Figure 3:**
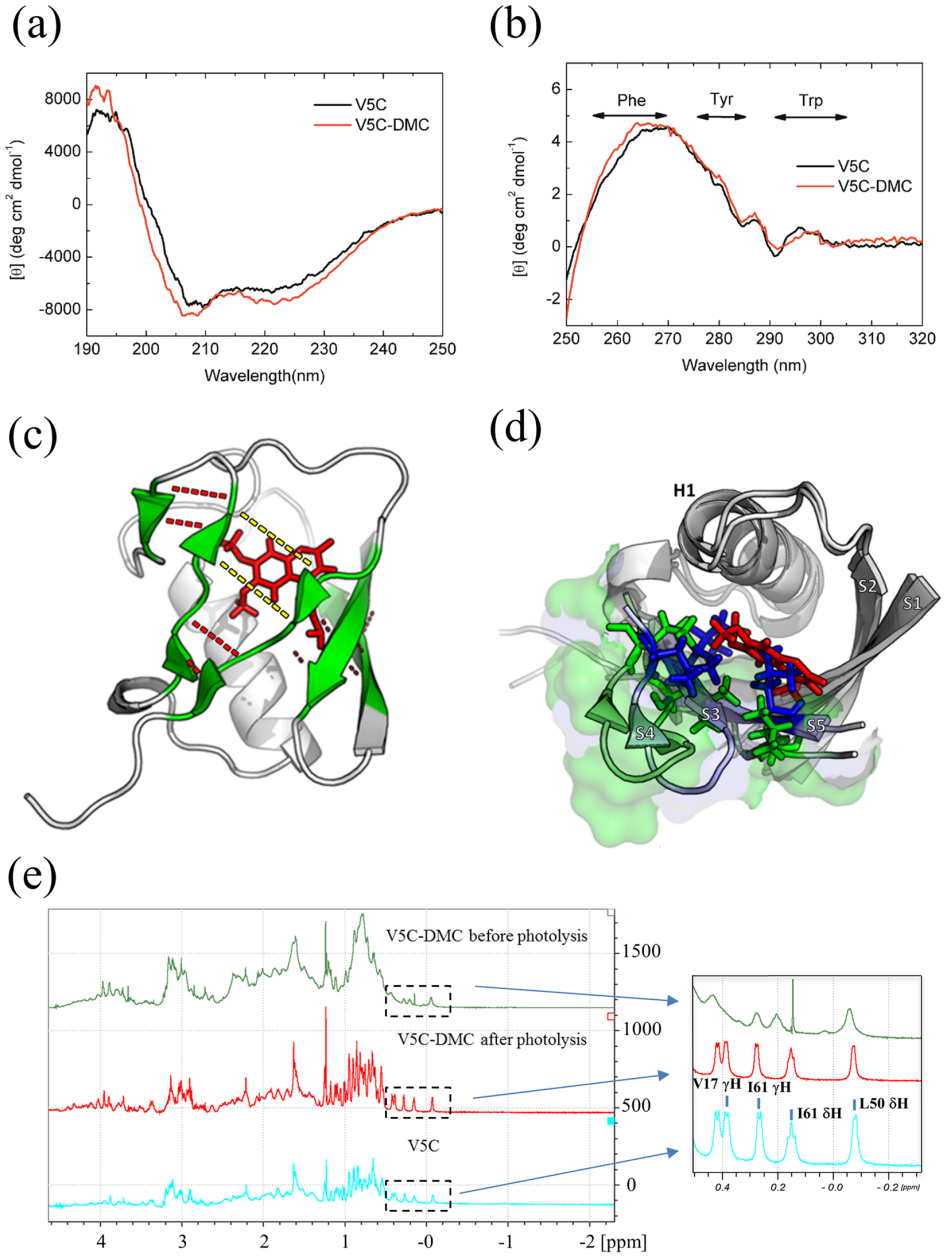
Structural comparison between V5C and V5C-DMC using CD, NMR and MD simulation. (**a**) Far-UV CD spectra of V5C and V5C-DMC. (**b**) Near-UV CD spectra of V5C and V5C-DMC. V5C and V5C-DMC were dissolved in DI (pH 5.7) to a final concentration of 4.76 and 6.59 μM for (**a**) and 40.9 and 44.2 μM for (**b**), respectively. (**c**) Hydrogen bonding networks in β-sheet of the simulated V5C-DMC structure. Red sticks: DMC. Green ribbons: β-strands. Red dotted lines: hydrogen bonds. Yellow dotted lines: hydrogen bonds lost in V5C-DMC but present in V5C. (**d**) Rearrangement of residues in the hydrophobic core of V5C-DMC. Green sticks: rearranged residues 43, 50 and 67 after simulation. Red sticks: DMC-conjugated Cys5 in the simulated V5C-DMC structure. Blue sticks: original positions of residues 43, 50 and 67 in V5C. If DMC was directly conjugated onto Cys5 without relaxing the structure, there is steric hindrance in residues 43, 50 and 67. The local protein surface was shown in light blue for V5C and light green for V5C-DMC. β-Strands are labeled as S1 - S5; helix 1 is labeled as H1. (**e**) Comparison of the 1D-NMR spectra of V5C (light blue), V5C-DMC before photolysis (green), and V5C-DMC after photolysis (red). The concentration of V5C and V5C-DMC is 175 and 20 μM, respectively. The concentration of photolytic product of V5C-DMC, i.e. refolded V5C, is 60 μM.

To clarify whether the β-sheet structure is disrupted by adding the DMC moiety, the secondary and tertiary structures of V5C and V5C-DMC were compared by circular dichroism spectroscopy. The far-UV CD spectra (Fig. 3a) showed that the secondary structure of V5C was slightly affected by the addition of the DMC group. The structure was changed back after the DMC is cleaved by photoreaction (see Supplementary Fig. S1). Deconvolution of CD spectra showed about 5% less β-sheet content for V5C-DMC than V5C (see Supplementary Table S1). Using homology modeling and all-atom MD simulation, the results showed that the cage moiety of the simulated V5C-DMC structure was buried inside the hydrophobic core of ubiquitin (Fig. 3c) and that V5C-DMC has a lower β-sheet content than V5C (see Supplementary Table S1). A superimposed image of V5C and V5C-DMC shows that the side chains of residues 43, 50, and 67 (Fig. 3d, blue residues) located on strands β3, β4, and β5, respectively, must rearrange in order to accommodate the DMC group. Moreover, comparing the hydrogen bonding network, the most prominent difference is the loss of two hydrogen bonds between residue 44 (on β3) and residue 68 (on β5) (Fig. 3c; see Supplementary Table S2). Although Val5 is situated on β1, the major structural change occurs at strands 3-5 upon DMC labeling as indicated by the residue rearrangement (Fig. 3d) and hydrogen bonding networks (Fig. 3c). The structural rearrangement leads to a volume expansion of about 178.8 Å^3^ per molecule by comparing the simulated structures of V5C and V5C-DMC (Fig. 3d).

The near-UV CD spectra were used to detect tertiary structure differences, because the aromatic residues (Phe4, Trp45, and Tyr59) are sensitive to their surrounding environment^39^. The near-UV CD spectra of V5C and V5C-DMC (Fig. 3b) showed significant difference in the tertiary structure around Phe4. Although these two near-UV CD spectra also showed difference in the range of 288–296 nm, we cannot conclude whether the tertiary structure around Trp45 is affected or not. It is because both Trp and DMC have absorption at 280 nm and we cannot conclude whether the difference is attributable to Trp or DMC.

NMR spectroscopy revealed further structural information about V5C and V5C-DMC (before and after photolysis). The 1D NMR spectrum of V5C-DMC (green) showed a clear difference in the high field region (0–0.5 ppm) when compared with the spectrum of V5C (light blue). Ubiquitin is a well packed protein and its protons on the aliphatic side-chain of the residues V17, L50, and I61 are shielded by the surrounding hydrophobic environment and hence upfield-shifted in the 1D-NMR spectrum (Fig. 3e). After modification with the cage, these upfield-shifted protons broadened and downfield shifted, suggesting that the tertiary structure of V5C-DMC is not as well-packed as V5C. The 1D spectrum of V5C-DMC after photolysis (red) is very similar to the 1D spectrum of V5C (light blue), suggesting that the protein can refold back to its native state after photolysis. Moreover, the comparison of 2D TOCSY spectra of V5C and V5C-DMC suggested that V5C-DMC aggregated in the experimental condition because higher protein concentration is required for NMR measurement (see Supplementary Fig. S2). The HN-Hα crosspeaks of I13, W45, E64, T66, L67, H68 disappeared in the spectra of V5C-DMC, suggesting that the hydrogen bond network of the β-sheet was affected and the residues on the β-sheet became more flexible. The data are consistent with the simulated results in which the hydrogen bonds between I44 (β3) and H68 (β5) were lost and the hydrogen bonds between strands β1 and β5 were rearranged (see Supplementary Table S2).

**Figure 4:**
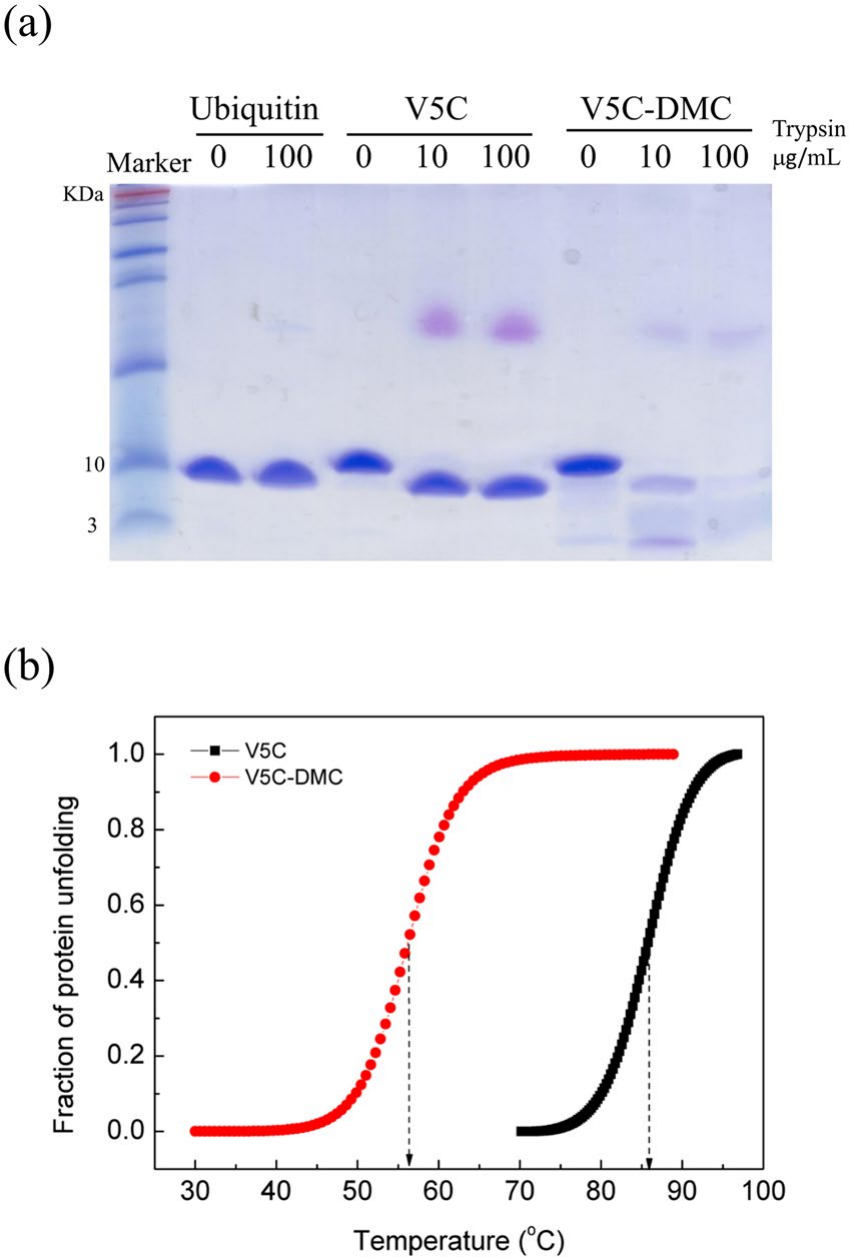
Comparison of structural stability of V5C and V5C-DMC. V5C and V5C-DMC were dissolved in DI (pH 5.7) to a final concentration of ~20 μM. (**a**) Wild-type ubiquitin, V5C, and V5C-DMC were digested by different concentrations of trypsin and analyzed by SDS-PAGE. (**b**) The thermal denaturation curves of V5C and V5C-DMC.

Although the overall structural differences induced by cage addition seem small, the structural compactness is largely affected. It is well known that ubiquitin is compact and protease-resistant. As shown by the trypsin digestion assay (Fig. 4a), V5C was as stable as wild-type ubiquitin against digestion by trypsin with the enzyme concentration as high as 100 μg/mL (though the C-terminal His-tag of V5C can be cleaved so that the band is shifted to a lower position), whereas V5C-DMC was digested easily even at low trypsin concentration (10 μg/mL). The decrease of structural stability by the DMC modification was further confirmed by the thermal denaturation experiment (Fig. 4b). The T_m_ of V5C-DMC is 56 °C, which is 29 °C lower than the T_m_ of V5C (85 °C). Based on the above data; we concluded that V5C-DMC has a non-native-like, partially misfolded/unfolded structure. The major structural difference occurred at the β-sheet, and this structural change affected protein stability and compactness.

### Time-resolved photothermal signals and deconvolution

**Figure 5:**
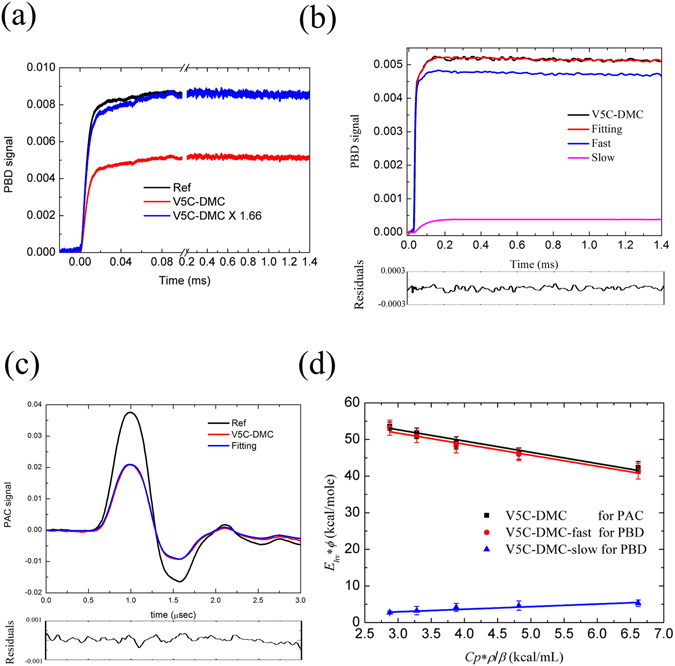
The refolding kinetic data of V5C-DMC after photolysis observed by PAC and PBD. (**a**) The PBD signals of reference (black), V5C-DMC (red) and V5C-DMC multiplied by 1.66 (blue). (**b**) The deconvolution results of PBD signals: V5C-DMC (black), the deconvoluted fast (blue) and slow (pink) events, and the fitting curve (red). (**c**) The PAC signals of reference (black), V5C-DMC (red), the fitting curve (blue). The corresponding residuals of fitting are shown below the plots. (**d**) The plots are of *E*_hv_*ϕ versus *C*_p_*ρ/β for the PAC signal (■), the fast event of PBD signal (V5C-DMC-fast, ●), and the slow event of PBD signal (V5C-DMC-slow, ▲).

The refolding process of V5C-DMC after photoirradiation was revealed by the time-resolved photothermal methods, photoacoustic calorimetry (PAC) and photothermal beam deflection (PBD). The PBD results are shown in Fig. 5a. The reference signal is shown in black and the sample signal is shown in red. To clearly see the difference between these two signals in the time range of 0–0.06 ms, the sample signal is multiplied 1.66-fold and is shown in blue. The slower response curve of the blue line suggested the existence of a slow event. Because the overall PBD signal is the convolution of all events plus the instrument response function, the photocleavage event and the following V5C refolding event in the PBD signal could be deconvoluted by equation (1) in the Methods section. Two events, fast (ϕ_PBD1_ ~ 0.589, τ_PBD1_ < 2 μs, blue line) and slow (ϕ_PBD2_ ~ 0.051, τ_PBD2_ ~ 60 μs, purple line), were resolved and are shown in Fig. 5b (The comparison of single and double exponential fitting is shown in Supplementary Fig. S3). The PBD signal comes from the refractive index change, which resulted from the heat release of photoinduced transient species, the volume change of the photoinduced sample, the absorption spectrum change of the sample after photoexcitation, and the optical Kerr effect^40^. To avoid the photoinduced absorption spectrum change, the probe laser wavelength should be far away from the absorption spectrum of the reactants, intermediates, and products of the sample after photoexcitation. Therefore, the He-Ne laser (632.8 nm) was used as a probe laser to avoid the phenomenon^41^. In addition, the Kerr effect occurs on the femtosecond to picosecond timescale, so it unlikely to contribute to the observed slow event^42^. Thus, the PBD signal mainly came from the heat release and volume change of V5C-DMC upon photoexcitation. In particular, the timescale of the slow event of PBD signal is about 60 μs, and it should represent the β-sheet refolding signal of V5C. The fast event, which is faster than 2 μs (PBD time limitation), was further analyzed by PAC (Fig. 5c). The PAC technique can monitor events occurring in the time range from 20 nanoseconds to 10 microseconds. The reference signal (black line) displays the instrument response function of PAC, and the red line shows the PAC signal of V5C-DMC upon photoexcitation. The red line was fitted using equation (1) and then shown as blue line. The line can be fitted by single exponential function to obtain the amplitude and time constant. The amplitude of ϕ_PAC1_ (0.590) is close to the amplitude of ϕ_PBD1_ (0.589), suggesting that the fast event observed by PBD is corresponded to the event observed by PAC, and its time constant is faster than 20 ns (PAC instrument response time). Because there was no other slow event that could be monitored by PAC, we can conclude that there is no refolding event occurring from 20 ns to 10 μs.

The amplitudes of PAC and PBD include the contributions of heat release and volume change and that can be further analyzed by Eq. (2)^40, 43^

2 $$E{}_{h\upsilon }\varphi _{i}={q}_{i}+{(\frac{{C}_{p}\rho }{\beta })}_{T}{{\rm{\Delta }}}_{R}{V}_{i}$$

where *E*_*hv*_ is the excitation energy (80.6 kcal/mol for 355 nm); *q*_*i*_ is the heat released in the system after excitation by 1 mole of photons; Δ_*R*_ *V*_*i*_ is the observed volume change in the *i*th process, Δ_*R*_ *V*_*i*_ = Φ_*c*_ Δ*V*_*i*_, where Φ_*c*_ is the photocleavage quantum yield and Δ*V*_*i*_ is the molar volume change of the solute in the *i*th process; *β* is the volumetric thermal expansion coefficient of the solvent; and *ρ* and *C*_*p*_ are the density and heat capacity of the solvent, respectively.

The PAC and PBD signals for V5C-DMC after laser irradiation were measured in the temperature range 15–35 °C. We used the same methods to deconvolute the data obtained. Using the *ϕ*_*i*_ obtained at different temperatures and equation (2), we calculated *q*_*i*_ and Δ_*R*_ *V*_*i*_ by linear fitting. The slope of the line corresponds to the observed volume change (Δ_*R*_ *V*_*i*_) of V5C-DMC after photocleavage, and the heat release value (*q*_*i*_) can be obtained from the intercept on the y-axis (Table 1). As shown in Fig. 5d, the fitting of the PAC data (black square) indicated that the observed volume change (Δ_*R*_ *V*) was −3.08 ± 0.32 mL/mol, and the heat released to the solution (*q*) was 61.89 ± 1.34 kcal/mol. For the PBD fast event (red circle), the observed volume change (Δ_*R*_ *V*_1_) was −3.01 ± 0.31 mL/mol, and the heat released to the solution (*q*_1_) was 60.80 ± 1.34 kcal/mol. Because the PAC event and PBD fast event had similar values for volume change and heat release, results from the two assays could be assigned as the same event. The fast event includes heat release and negative volume change from the above analysis, and we concluded that the event was the initial collapse of the protein after the photoexcitation. The collapse could probably be driven by hydrophobic interactions. For the PBD slow event (blue triangle), the observed volume change (Δ_*R*_ *V*_2_) was 0.71 ± 0.11 mL/mol, and the heat released to the solution (*q*_2_) was 0.81 ± 0.41 kcal/mol (Fig. 5d).

**Table 1:** The data of thermal and volume change obtained from PAC and PBD. F and S denote the fast and the slow events, respectively.

V5C-DMC	Heat release (kcal/mol)	Enthalpy change (kcal/mol)	Volume change (mL/mol)
PAC Event	61.89 ± 1.34	−208.74 ± 89.34	−3.08 ± 0.32
PBD Event F	60.80 ± 1.34	−136.73 ± 89.34	−3.01 ± 0.31
PBD Event S	0.81 ± 0.41	−54.00 ± 27.33	0.71 ± 0.11

**Figure 6:**
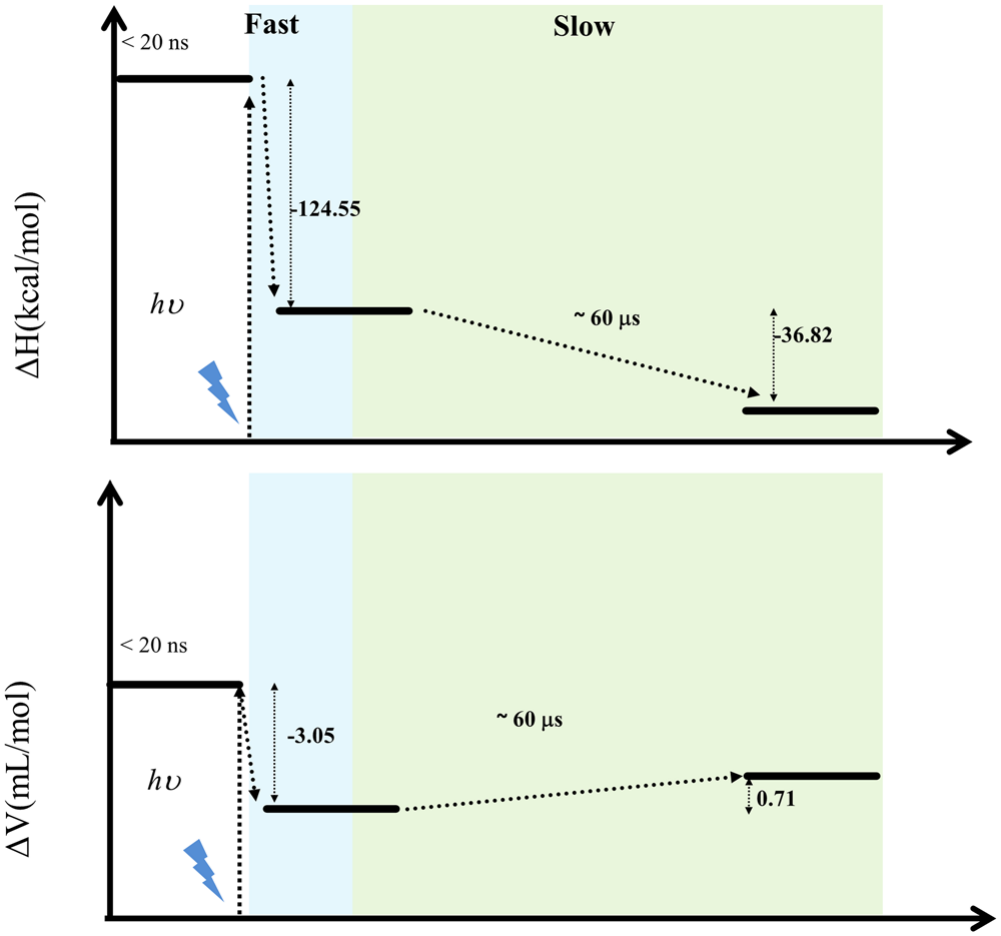
Refolding reaction coordinate of the partially misfolded V5C-DMC after photolysis.

In our previous work on RD1 refolding by the same caging strategy^29^, the photocleavage yield was estimated based on caged peptide due to the similarity between caged RD1 and caged peptide in the solvent accessibility of the cage. However, DMC is shown to be buried inside and quite isolated from water molecules in the case of V5C-DMC. The chemical yield of the cage release process, which requires the attack of a photoexcited hydroxyl radical on DMC, would be expected to be quite low and cannot be adapted from previous estimation. To reasonably estimate the chemical yield, it is possible to infer from the volume difference between DMC-labeled and unlabeled V5C using the equation: chemical yield = ∆V_observed_/∆V_theoretical_. As calculated using Chimera with solvent probes of 1.4 Å radius, the volume gain of V5C-DMC would be 178.8 Å^3^ per molecule (Fig. 3d). With the observed overall volume change of −2.34 mL/mole (−3.05 mL/mole, which is the average of PAC and fast PBD event, plus 0.71 mL/mole in the slow PBD event), which corresponds to 3.89 Å^3^ per molecule, the chemical yield of cage release is ~2.2%, which is about 10 times smaller than the cleavage quantum yield measured for the DMC-labeled peptide in our previous study^29^. Once we obtained the chemical yield, we were then able to calculate the thermal release of DMC upon photon excitation and the enthalpy change of proteins. Based on the energy conservation theory and fluorescence quantum yield (see Supplementary Fig. S4), the enthalpy was −124.55 ± 60.91 kcal/mol and −36.82 ± 18.64 kcal/mol for the fast and slow events, respectively (Fig. 6)^29^. Both events are exothermic processes, and the system releases heat to its surroundings.

## Discussion

The V5C-DMC folding process is illustrated in Fig. 6. According to the simulated V5C-DMC structure, DMC is buried inside the hydrophobic core of ubiquitin. Therefore, V5C-DMC is still soluble (compared with DMC modified RD1, which is prone to aggregation), but is less stable. After the cage is released upon photoirradiation, the misfolded structure is no longer stable and spontaneously refolds, which corresponds to the fast event recorded in PBD. Since the timescale of hydrophobic collapse is from about one hundred nanoseconds to a few hundred microseconds, depending on the characteristics of the target protein and the detection methods^14, 44–47^, we propose that the fast compression of the protein is driven by hydrophobic force.

In contrast, the slow event shows a very small volume expansion (ΔV_2_ = 0.71 ± 0.11 mL/mol), and the timescale of refolding is about 60 μs. Near-UV CD spectroscopy and computer simulation suggested that the misfolded part is primarily at the N-terminal strand β1 and the C-terminal strands β3 and β5. The slow event is hence considered as a structural rearrangement which involves reformation of hydrogen bonds among β1, β3, and β5.

Previously, we employed the same strategy to study the refolding kinetics of RD1. The clarity of our results prompted similar investigation into a more complex protein, though there were differences between these two studies, including: (1) RD1 is a small protein possessing only several short pieces of secondary structures, whereas ubiquitin is a very stable protein with a high percentage of secondary structures; (2) The DMC coupling site on RD1 is in the interior of the protein, whereas the DMC coupling site in ubiquitin is quite solvent exposed; (3) Due to the selection of the DMC coupling site, DMC completely unfolded RD1, whereas DMC only induced a partially misfolded structure in ubiquitin; (4) Because DMC-coupled RD1 is unfolded and prone to aggregation in water, the kinetic measurement can only be done in the presence of GdnHCl, whereas the refolding of DMC-coupled ubiquitin can be recorded in water. Selecting Val-5 of ubiquitin as the DMC coupling site enabled us to monitor the protein refolding kinetics in the absence of any denaturants.

Shaw and his colleagues studied the equilibrium folding/unfolding kinetics by all-atom MD simulation^48^. Their study reported a near-native misfolded state (MF3) with a refolding timescale of 18 μs. The time is in the same scale as the rearrangement timescale of the misfolded ubiquitin in our measurement (60 μs).

It is known that a protein can partially unfold under certain conditions. The partially unfolded protein might expose its hydrophobic segment and this exposed segment is then prone to association due to hydrophobic interaction. In most cases, the partially unfolded proteins associate with each other and form amorphous aggregates. Some proteins, such as prion protein, α-synuclein, and insulin, and peptides such as Aβ peptides can associate into oligomers and amyloid fibrils, leading to various diseases^3^. Despite such severe consequences, the partially unfolded state is rarely studied due to difficulties arising from its instability. It either quickly converts back to native state or forms aggregates (Fig. 1). Therefore, it is difficult to study its structure or refolding kinetics. In this study, we artificially locked ubiquitin in a partially unfolded state using a photolabile cage so that we could assess conformational differences. We then used photo-irradiation to release the cage and monitored the refolding process of ubiquitin from this partially unfolded state. Our data indicated that the refolding of a β-sheet in a protein is slower than β-hairpin or β-sheet formation from a peptide. It might be because the locally misfolded portion has tertiary contact with other parts of the protein, and the polypeptide chain therefore could not freely diffuse or rotate to find the native-state. It has been proposed that the intermediate state could lead to less efficient folding^49^. We surmise that this internal friction of side-chains when a protein forms the hydrophobic core by hydrophobic force is the origin of the energy barrier.

## Conclusions

The local β-sheet refolding process of V5C-DMC has been clearly described by combining experiments and simulation. Our results showed that the refolding time of a protein with a misfolded β-sheet structure is longer than that required for the *ab initio* folding of a β-sheet peptide. Our data provides a missing piece of the puzzle in the protein folding landscape.

## Electronic supplementary material

Directly monitor protein rearrangement on a nanosecond-to-millisecond time-scale
