# Supplementary material for: Directly monitor protein rearrangement on a nanosecond-to-millisecond time-scale
Source: Sci Rep. 2017 Aug 18;7:8691. doi: 10.1038/s41598-017-08385-0 (PMC5562898; doi:10.1038/s41598-017-08385-0)
Supplement: Supplementary file 1 — Directly monitor protein rearrangement on a nanosecond-to-millisecond time-scale [file 41598_2017_8385_MOESM1_ESM.doc]

Directly monitor protein rearrangement on a nanosecond-to-millisecond time-scale

Eric H.-L. Chen^1^, Tony T.-Y. Lu^1,2^, Jack C.-C. Hsu^1^, Yufeng Jane Tseng^3,4^, T.-S. Lim^5^, Rita P.-Y. Chen^1,2*^

^1^Institute of Biological Chemistry, Academia Sinica, Taipei, 115, Taiwan

^2^Institute of Biochemical Sciences, National Taiwan University, Taipei 106, Taiwan

^3^Department of Computer Science and Information Engineering, National Taiwan University, Taipei 106, Taiwan

^4^Graduate Institute of Biomedical Electronics and Bioinformatics, National Taiwan University, Taipei 106, Taiwan

^5^Department of Physics, Tunghai University, Taichung 407, Taiwan

**Supplementary information**

Table S1. Comparison of secondary structure content between V5C and V5C-DMC obtained by simulation and far-UV CD spectrum.

| **Name Content(%)** | | **α-helix** | **β-sheet** | **Other** |
| --- | --- | --- | --- | --- |
| **Simulation** | V5C | 0.21 | 0.30 | 0.49 |
|  | V5C-DMC | 0.21 | 0.17 | 0.52 |
| **Experiment** | V5C | 0.22 | 0.27 | 0.51 |
|  | V5C-DMC | 0.23 | 0.22 | 0.55 |

Table S2. Number of hydrogen bonds in the β-sheets of V5C and V5C-DMC.

| Location | Number of hydrogen bonds^a^ | | |
| --- | --- | --- | --- |
|  | V5C-DMC | V5C | Difference |
| β4-β3 | 1.921^b^ | 1.915 | 0 |
| β3-β5 | 1.803^c^ | 3.774 | -2^d^ |
| β5-β1 | 1.904^e^ | 2.012^f^ | 0^g^ |
| β1-β2 | 3.898 | 3.830 | 0 |

^a^ H-bond numbers were calculated by averaging the numbers of H-bonds throughout the simulation snapshots

^b^ H-bond pairs: W45-N/K48-O, K48-N/ W45-O

^c^ H-bond pairs: R42-N/V70-O, V70-N/R42-O

^d^ Lost H-bonds: I44-N/H68-O, H68-N/I44-O

^e^ H-bond pairs: K6-N/L67-O, L69-N/K6-O

^f^ H-bond pairs: K6-N/L67-O, L67-N/F4-O

^g^ The H-bond between L67-N/F4-O disappears and the H-bond between L69-N/K6-O is formed after DMC modification.

(a)


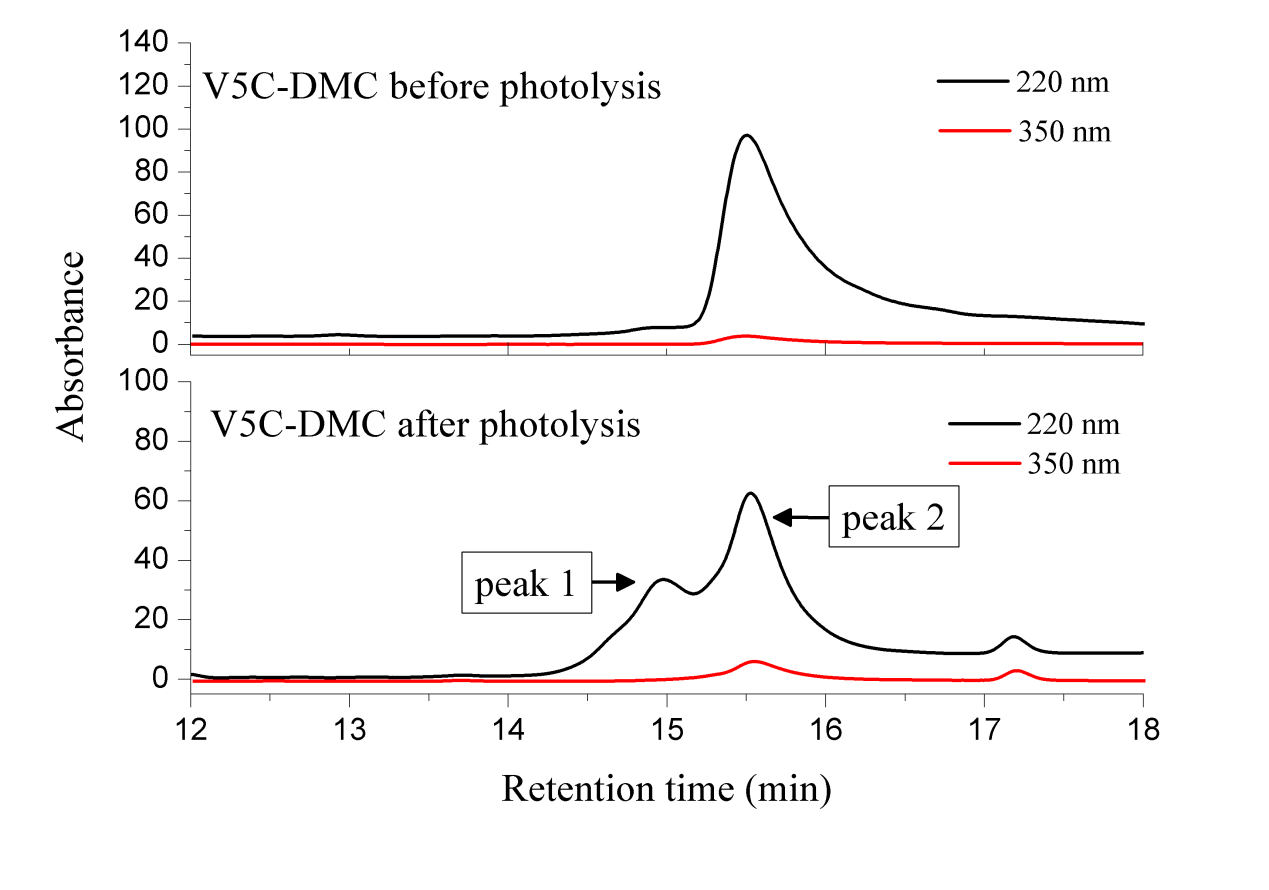


(b)


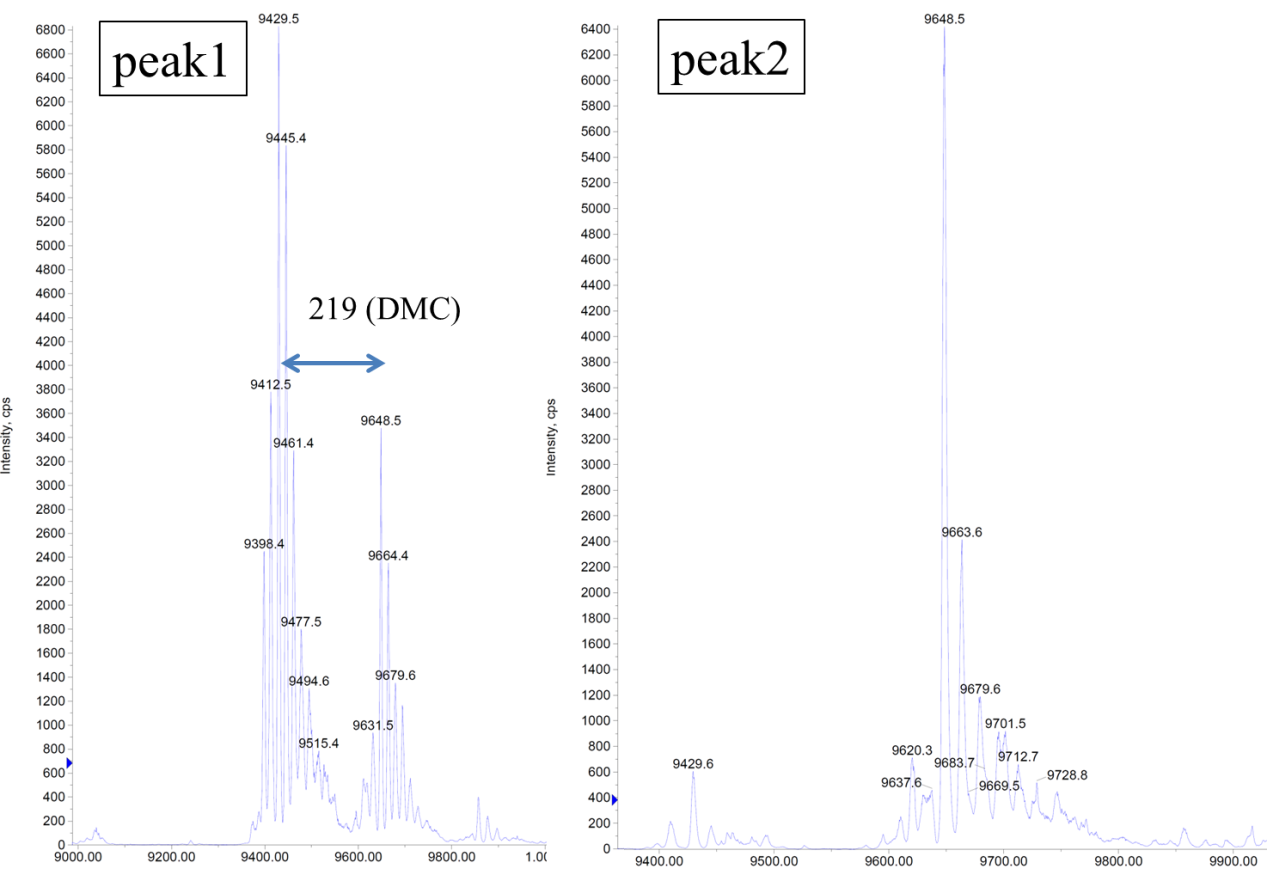


(c)


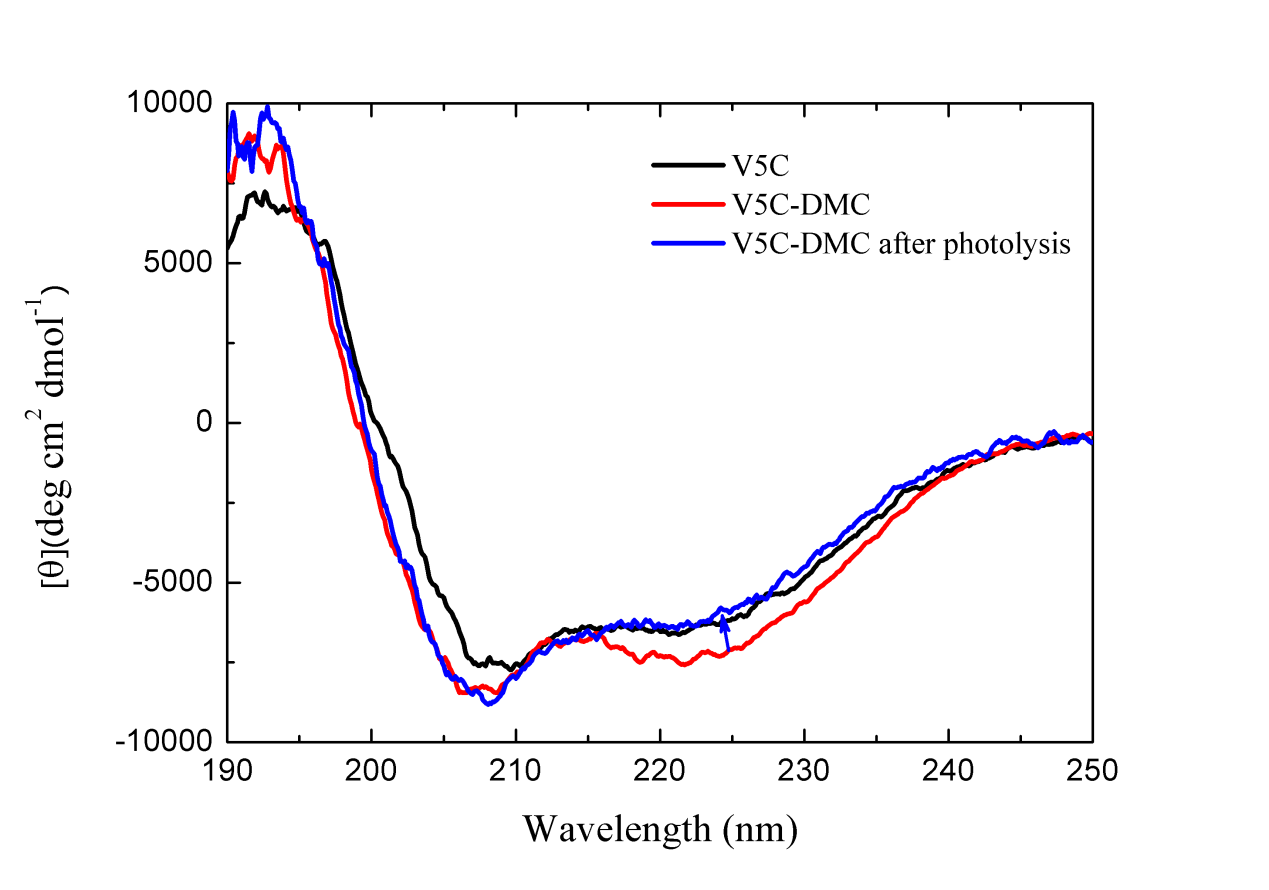


Figure S1. Photolysis analysis of V5C-DMC. (a) The HPLC chromatogram of V5C-DMC before and after photolysis. V5C-DMC was dissolved in DI, photolyzed at 352 nm (power 8W) in a photoreactor PR-2000, Panchum Scientific Corp. for 1 min, then analyzed by HPLC. The column is SUPELCO Discovery® Bio Wide Pore C18. Buffer A: 5% acetonitrile/0.1% TFA in DI; Buffer B: 0.1% TFA in acetonitrile. The elution was conducted by linearly increasing Buffer B from 0 to 100% in 30 min. The products were detected by monitoring the absorbance at 220 and 350 nm. Two peaks can be identified after photolysis. Peak 1 is V5C from photolyzed V5C-DMC and peak 2 is uncleaved V5C-DMC. (b) Mass spectra of peak 1 and peak 2. The theoretical mass of V5C and V5C-DMC is 9429 and 9648 Da, respectively. (c) CD spectra of V5C (black), V5C-DMC (red), and V5C-DMC after 1-min photolysis (blue). The spectrum shifting of V5C-DMC after photo-irradiation occurs in the region of 210-250 nm and is indicated by a blue arrow. The spectral change proves that photolyzed V5C-DMC refolds back to the structure of V5C. V5C-DMC and photolyzed V5C-DMC are different from V5C in the spectra ranged between 190-210 nm due to the contribution of DMC.

Figure S2. The 2D TOCSY spectra of V5C (red) and V5C-DMC (green).

**(a)**

**
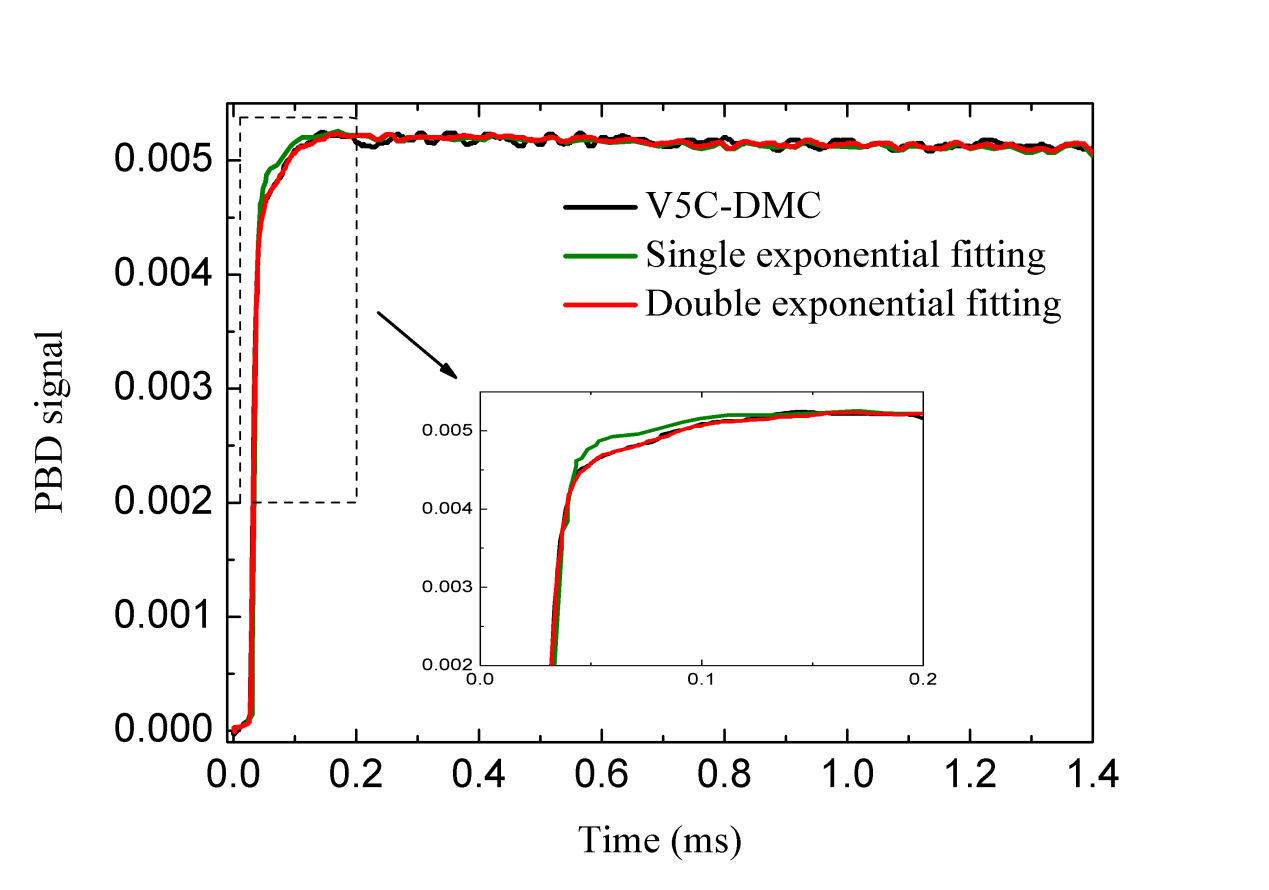
**

**(b)**

**
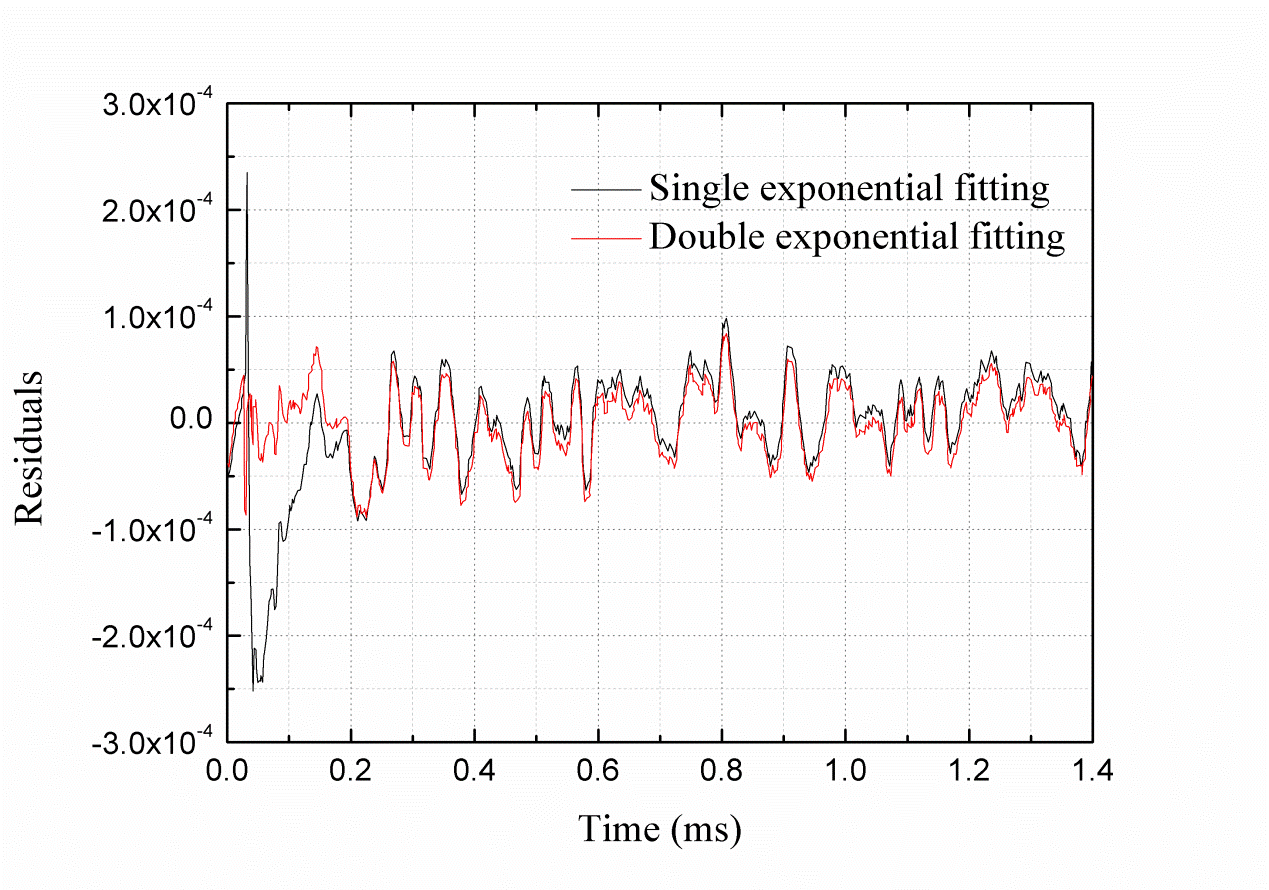
**

Figure S3. Fitting analysis of PBD signals by single or double exponential fitting. (a) The PBD signals and the fitting curves of V5C-DMC after laser photolysis. The refolding kinetics of V5C-DMC after laser photolysis was recorded by PBD at 25 °C. Black line: observed signals; green line: fitting curve by single exponential equation; blue line: fitting curve by double exponential equation. (b) Residuals obtained from single or double exponential fitting of the PBD results. The residuals obtained by single exponential fitting are much bigger than those obtained by double exponential fitting at the time window of 40-200 μs.


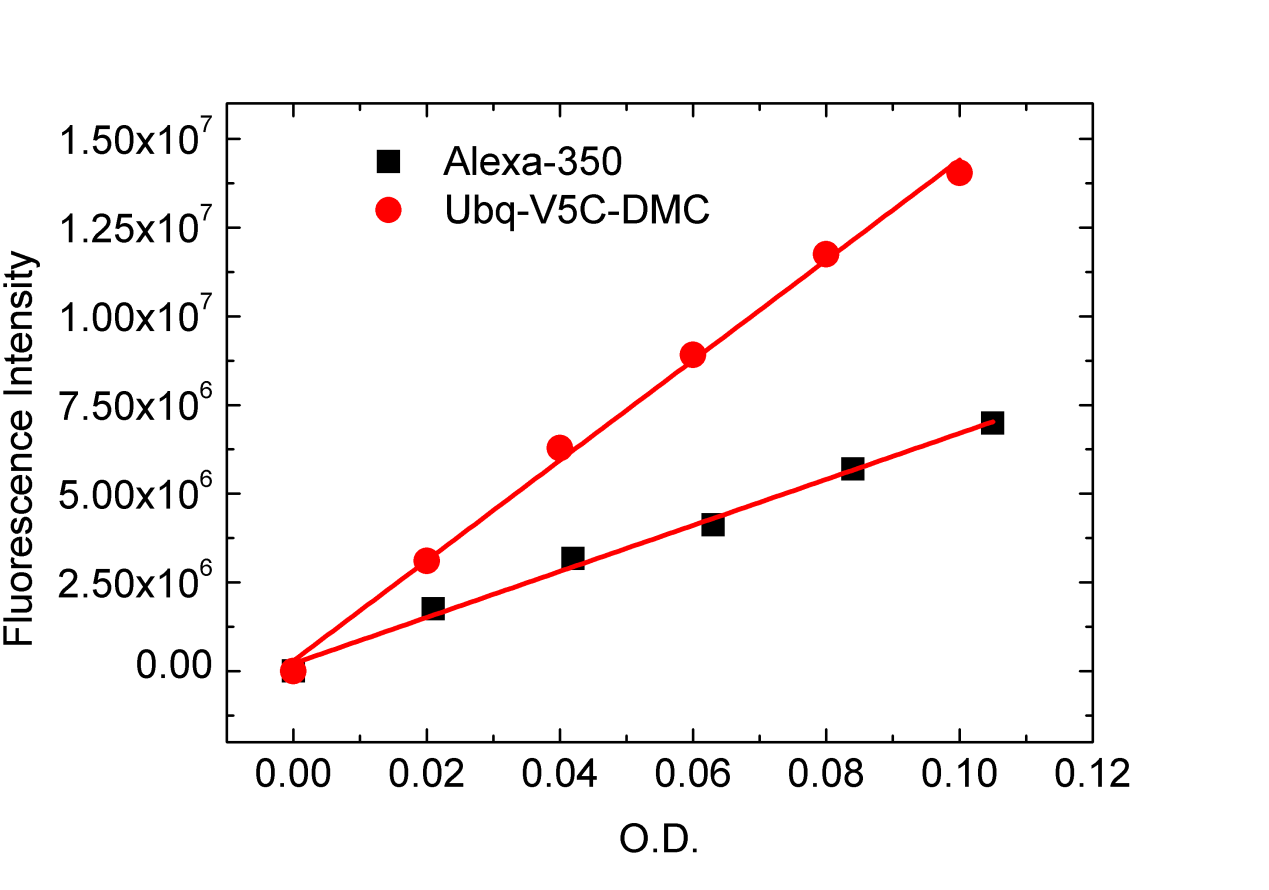


Figure S4. Fluorescence intensity of different concentrations of Alexa-350 and V5C-DMC. The concentrations of dye and protein are expressed as absorbance at 350 nm. Samples were excited at 350 nm and fluorescence spectra between 360 nm to 600 nm were recorded. The fluorescence intensity was obtained by integrating the centered at 440 nm in the fluorescence spectrum.

The fluorescence quantum yield of V5C-DMC was measured at various protein concentrations on a fluorescence spectrometer.^1^ Figure S1 shows the plot of the integrated fluorescence intensity versus absorbance (to avoid self-absorption, the O.D. should be close to, or lower than, 0.1). The slope S for each sample is proportional to its fluorescence quantum yield, which follows the relation: Φ_x_ = Φ_r_ (S_x_/S_r_)(n_x_/n_r_)^2^, where n represents the refractive index of the solvent adopted for each measurement and subscripts r and x denote the reference and sample, respectively. Using Alexa-350 as reference (Φ_f_ = 0.15), the fluorescence quantum yield of V5C-DMC was calculated as 0.33.

**Supplementary methods**

*MD simulation parameters*

All simulations were performed using the leapfrog algorithm to integrate Newton’s equations of motion with 0.5 fs time step. Neighbor searching was conducted using the Verlet algorithm with the pair list updated every 20 steps under periodic boundary condition. Electrostatic interactions were calculated using the PME method with cutoff at 0.9 nm. Van der Waals interactions were calculated under the cut-off scheme with cutoff at 0.9 nm. All bonds were constrained using the LINCS algorithm. A nose-hoover thermostat was used for temperature coupling at 25°C (298K) with the time constant set to 0.2 ps. A Parrinello-Rahman barostat was used for pressure coupling at 1 atmospheric pressure with the time constant set to 1 ps.

*Partial unfolding of V5C*

The structure of V5C was first solvated in a box with the size of 8 nm and 16616 TIP-3P water molecules.^2^ The protonation state for each residue was at neutral pH, leaving a net charge of 0. The system was first energy minimized using the steepest descent method with position restraints followed by equilibrium at 25°C (298K) NVT for 50 ps and then at 25°C (298K) NPT for 50 ps.

The equilibrated structure was then subjected to partial unfolding by two phases of heating. During the first phase, the system was heated from 25°C (298K) to 227°C (500K) for the first 50 ps and equilibrated for the remaining 950 ps. For the second phase, solutes were kept at 227°C to avoid dramatic changes in water density while the protein was heated from 227°C (500K) to 527°C (800K) for the first 75 ps and started to unfold for the remaining 400 ps. When the V5C site was solvent-exposed enough to accommodate a DMC molecule, the structure was snapshotted and conjugated with DMC using Chimera.

*Force field parameters of DMC*

To calculate the force field parameters of the modified amino acid, cysteine-DMC, the N- and C-end capped cysteine-DMC (an acetyl group and a methylamino group was linked to the N- and C-terminus, respectively, to mimic peptide bonds) was built with Chimera. The resulting structure was energy minimized and processed by AmberTools 14 to generate parameters via quantum chemical calculations. Preliminary parameters were mapped to the parameters in Amber99SB-ILDN force field by linear regression before use.

**References**

(1) Williams ATR, Winfield SA, Miller JN. Relative fluorescence quantum yields using a computer-controlled luminescence spectrometer. *Analyst*, **108**, 1067-1071 (1983).

(2) Jorgensen, W. L.; Chandrasekhar, J.; Madura, J. D.; Impey, R. W.; Klein, M. L. Comparison of simple potential functions for simulating liquid water. *J. Chem. Phys.*, ***79***, 926-935 (1983).
